# Genetic Features of HIV-1 Integrase Sub-Subtype A6 Predominant in Russia and Predicted Susceptibility to INSTIs

**DOI:** 10.3390/v12080838

**Published:** 2020-07-31

**Authors:** Alina Kirichenko, Ilya Lapovok, Pavel Baryshev, David A. M. C. van de Vijver, Jeroen J. A. van Kampen, Charles A. B. Boucher, Dimitrios Paraskevis, Dmitry Kireev

**Affiliations:** 1 Central Research Institute of Epidemiology, 111123 Moscow, Russia; i_lapovok@mail.ru (I.L.); pavel.fj@yandex.ru (P.B.); dmitkireev@gmail.com (D.K.); 2 Viroscience Department, Erasmus Medical Centre, 3015 CE Rotterdam, The Netherlands; d.vandevijver@erasmusmc.nl (D.A.M.C.v.d.V.); j.vankampen@erasmusmc.nl (J.J.A.v.K.); c.boucher@erasmusmc.nl (C.A.B.B.); 3 Department of Hygiene, Epidemiology and Medical Statistics, Medical School, National and Kapodistrian University of Athens, 11527 Goudi, Athens, Greece; dparask@med.uoa.gr

**Keywords:** HIV-1, A6, integrase, INSTI, L74I, polymorphisms, genetic barrier, founder effect, phylogenetics, Russia

## Abstract

The increasing use of the integrase strand transfer inhibitor (INSTI) class for the treatment of HIV-infection has pointed to the importance of analyzing the features of HIV-1 subtypes for an improved understanding of viral genetic variability in the occurrence of drug resistance (DR). In this study, we have described the prevalence of INSTI DR in a Russian cohort and the genetic features of HIV-1 integrase sub-subtype A6. We included 408 HIV infected patients who were not exposed to INSTI. Drug resistance mutations (DRMs) were detected among 1.3% of ART-naïve patients and among 2.7% of INSTI-naïve patients. The prevalence of 12 polymorphic mutations was significantly different between sub-subtypes A6 and A1. Analysis of the genetic barriers determined two positions in which subtype A (A1 and A6) showed a higher genetic barrier (G140C and V151I) compared with subtype B, and one position in which subtypes A1 and B displayed a higher genetic barrier (L74M and L74I) than sub-subtype A6. Additionally, we confirmed that the L74I mutation was selected at the early stage of the epidemic and subsequently spread as a founder effect in Russia. Our data have added to the overall understanding of the genetic features of sub-subtype A6 in the context of drug resistance.

## 1. Introduction

It was estimated that more than 1 million individuals were living with HIV-1 in the Russian Federation at the end of 2019, which is 0.7% of the total population of the country [1].

Advances in combination antiretroviral therapy (cART) have improved treatment effectiveness for people living with HIV, increasing life expectancy and quality of life.

The HIV-1 integrase strand transfer inhibitor (INSTI) class of antiretroviral drugs is the latest to be approved for treatment and is the favored class recommended by different international guidelines, including the Russian treatment guidelines since 2017, as part of first-line treatment and salvage regimens [2,3,4,5,6].

Both clinical trials and real-life data have demonstrated the high efficacy, favorable safety profile, tolerability, low toxicity, and relatively high genetic barrier to resistance of INSTIs for both naïve and ART-experienced patients [7,8,9].

As the name suggests, INSTIs inhibit the second step of viral replication catalyzed by integrase, i.e., strand transfer, through competitive binding to the enzyme’s active site. HIV-1 integrase has three independent domains: The N-terminal domain, the catalytic core domain, and the C-terminal domain. Each region comprises motifs essential for the proper functioning of the enzyme, e.g., the conserved zinc finger motif (H12-H16-C40-C43) in the N-terminal domain, the active site (D64-D116-E153) in the catalytic core domain, and the minimal nonspecific DNA-binding region ranging from I220 to D270 in the C-terminal domain [9,10,11,12,13,14].

There are currently four INSTIs approved by the Food and Drug Administration (FDA) for the treatment of HIV infection: Raltegravir (RAL), elvitegravir (EVG), dolutegravir (DTG), and bictegravir (BIC). RAL was the first INSTI drug approved for the treatment of HIV-infected patients in Russia in 2008, followed by DTG and EVG in 2014 and 2019, respectively. RAL and DTG have been included in the Vital and Essential Drugs guidelines and have been entered as the preferred first-line treatment regimen [6].

Another INSTI, cabotegravir (CAB), is the newest drug that is being developed as a long-acting injectable for monthly or quarterly administration and as an oral tablet for daily use for the treatment and prevention of HIV-1 infection, and it is currently in the late phase of clinical trials [8,15]. At present, a long-acting regimen with CAB has been approved by Health Canada. Recently, the FLAIR clinical trial showed that a long-acting regimen of CAB and rilpivirine (RPV) is noninferior for HIV maintenance therapy compared to DTG, abacavir (ABC), and lamivudine (3TC) in naïve patients. However, 3 participants experienced virological failure. Interestingly, these patients were all from Russia and had an HIV infection with subtype A and the L74I mutation before the treatment initiation [15]. The efficacy outcomes in this study were similar to those of the ATLAS study, which had 2 treatment experience participants with the L74I mutation from the Russian Federation with virological failure after therapy containing CAB [16].

The L74I substitution in integrase has been described as a polymorphic mutation in a low but substantial proportion (21%) of subtype A sequences [17]; however, according to previous studies in Russia and the former Soviet Union (FSU) countries, the prevalence of this mutation varied from 93% to 100% [18,19,20,21].

The L74I mutation is located in the catalytic core domain and is part of the hydrophobic cluster including L63, T97, F100/Y100, L101, L113/I113, and F121 near the active site of integrase [22]. In vitro selection experiments indicated that substitutions in the 74 position combined with major mutations (G140A/C/S, Q148 H/K/R) and other accessory mutations (V75I, T97A) can significantly reduce susceptibility to INSTI, whereas this mutation alone has minimal impact on INSTI susceptibility or HIV-1 replication capacity [7,23,24,25,26,27,28,29,30].

Thus, the role of substitutions in the 74 position, as well as other polymorphisms, in the development of resistance remains the subject of speculation. Obviously, HIV-1 polymorphisms do not always confer antiretroviral resistance directly, and the possible mechanisms by which polymorphisms affect drug resistance mutation (DRM) emergence are diverse.

Moreover, data about the role of DRMs in INSTI and about the natural variability of integrase are predominantly available for viruses of subtype B, which represents less than 10% of the globally circulating viruses. However, because of the high variability in codon usage between subtypes, particular HIV-1 subtypes could have different genetic barriers for DRMs [31,32,33].

Recently, several studies of subtype-specific differences in sensitivity and the impact of naturally occurring polymorphisms were presented. Investigated polymorphic substitutions, especially at the active site of integrase, demonstrated that the natural variability of integrase across HIV-1 genetic variants can significantly affect the genetic barrier to drug resistance (DR) by influencing the selection of resistance mutations, native protein activity, structure, and the function of the drug-mediated inhibition of the enzyme, which may have important implications for INSTI therapy [13,22,34,35,36,37,38,39,40].

In Russia and FSU countries, the HIV-1 epidemic is dominated by sub-subtype A6 (formerly FSU-A or IDU-A) [41,42,43], and data about mutations, particularly natural polymorphisms in the integrase gene, are sparse and not well investigated for this viral clade [18,19,20,21].

Therefore, the expanded use of INSTIs in Russia makes it urgent to analyze the HIV-1 integrase gene. Following this need, the aims of this study were to describe INSTI resistance profiles among INSTI-naïve patients, to analyze the prevalence of naturally occurring polymorphisms and genetic barriers, and also to investigate the dispersal patterns of A6 resistance mutations by means of phylogenetic and phylodynamic analysis.

## 2. Materials and Methods

### 2.1. Study Population

We analyzed integrase sequences that had been obtained as part of routine clinical care from 408 HIV-infected patients between 2007 and 2019 in Russia; 225 treatment-naïve patients and 183 INSTI-naïve patients with virological failure to cART were included. This study was approved by the Ethics Review Committee of the Central Research Institute of Epidemiology (Moscow, Russia).

### 2.2. RNA Extraction and Sequencing Method

An AmpliSens^®^ HIV-Resist-Seq kit (Central Research Institute of Epidemiology, Moscow, Russia) was used for RNA extraction from the plasma samples and sequencing of the HIV pol-gene region coding integrase (4230–5093 bp according to HXB-2, GenBank accession number K03455).

### 2.3. HIV-1 Subtyping

HIV-1 subtypes were determined using integrase sequences by the Stanford HIV Resistance Database (https://hivdb.stanford.edu/) and subsequently clarified by phylogenetic analysis.

### 2.4. HIV-1 INSTI Resistance Profile Genotyping

The Stanford HIV Resistance Database (HIVdb Program v 8.9-1 and Calibrated Population Resistance Tool) was used to describe and interpret the INSTI resistance profiles.

### 2.5. Polymorphism Analysis

For this analysis, we used the treatment-naïve A6 sequences obtained in this study and worldwide HIV-1 treatment-naïve A1 sequences (*n* = 100) (4230–5093 bp) obtained from the Los Alamos HIV Database (www.hiv.lanl.gov accessed 23 March 2020).

Naturally occurring polymorphisms of subtype were defined as any substitution that occurred with a frequency of ≥1% in sequences of this subtype compared with the HXB2 HIV-1 clade B sequence (GenBank accession number K03455). All other positions were defined as conserved. Highly polymorphic positions were defined as positions that had substitutions detected in more than 50% of sequences of this subtype.

Fisher’s exact test was used to compare the proportion of amino acid substitutions between groups. Statistical significance was defined as *p*-values < 0.05.

### 2.6. Genetic Barrier Analysis

For this analysis, we used the treatment-naïve A6 sequences obtained in this study and worldwide HIV-1 treatment-naïve A1 sequences (*n* = 100) and B sequences (*n* = 2577) obtained from the Los Alamos HIV Database (www.hiv.lanl.gov accessed 23 March 2020). The calculations of genetic barriers were performed as published previously [32]. According to A.M. Vandamme [44] the transitions (replacement A→G and C↔T) occur 2.5 times more frequently than transversions (replacement A↔C, A↔T, G↔C, G↔T). Therefore, transitions were scored as 1 and transversions were scored as 2.5. Because of the high rate of APOBEC-mediated hypermutations (G to A) this type of transition was scored as 0.2 [45]. The missing nucleotides were scored as −777. Python v 3.7 script was used to score the genetic barrier to the drug resistance-associated mutations. The genetic barrier was the sum of the scores for each studied amino acid position.

Pairwise differences between subtypes A1, A6, and B sequences were analyzed with the Mann–Whitney test and the Benjamini–Hochberg method to correct for multiple hypothesis testing.

### 2.7. Phylogenetic and Phylodynamic Analysis

For this analysis, we used the A6 sequences obtained in this study; worldwide HIV-1 A sequences (*n* = 58), A1 sequences (*n* = 319), A2 sequences (*n* = 7), A3 sequences (*n* = 9), A4 sequences (*n* = 3), and A6 sequences (*n* = 370) obtained from the Los Alamos HIV Database (www.hiv.lanl.gov accessed 23 March 2020).

Phylogenetic trees were estimated from the underlying nucleotide sequences using the approximate maximum likelihood (ML) method with bootstrap evaluation under the generalized time reversible (GTR) model as a nucleotide substitution model—including a Γ distributed rate of heterogeneity among sites—as implemented in RaxML [46]. Further phylogenetic analysis was performed in FastTree v2.1 [47] to verify our results. Tree visualization and annotation were performed using FigTree v1.4.2 and iTOL (https://itol.embl.de/).

The phylodynamic analysis was conducted using a Bayesian approach as implemented in BEAST v2.2 [48]. We analyzed sequences found within the Russian samples by using the GTR as the nucleotide substitution model with gamma heterogeneity and an uncorrelated relaxed clock model with lognormal distribution. A Markov chain Monte Carlo (MCMC) analysis was run for 25 × 106 generations and sampled every 5000 steps, with the first 10% of samples being discarded as burn-in. The MCMC convergence and effective sample sizes (ESS) were confirmed using Tracer v1.5. A consensus tree was built, and the distribution was assessed from the posterior tree using TreeAnnotator v1.8 23.

### 2.8. GenBank Accession Numbers

The HIV-1 integrase sequences generated from this study are available in the NCBI database with GenBank accession numbers MT382663–MT383070.

## 3. Results

### 3.1. Study Population

We studied the plasma samples of 408 HIV-1-infected patients from different regions of Russia. The main characteristics (demographic, clinical, and laboratory data) of the study population are described in Table S1. The average age of the patients was 32 years (IQR 26–40 years), and the majority of patients were male (57.6%). The dominant transmission routes were heterosexual contact (40.9%), intravenous drug use (IDU, 25.0%), mother-to-child transmission (MTCT, 4.9%), and men who have sex with men (MSM, 5.6%).

### 3.2. HIV-1 Subtyping

Online genotyping and subsequent phylogenetic analysis showed that the most frequent clade was sub-subtype A6 (350 sequences; 85.8%). The viral genetic distribution of other sequences was B (29; 7.1%), CRF63_02A1 (14; 3.4%), CRF02_AG (10; 2.5%), G (3; 0.7%), A1 (1; 0.2%), and CRF03_AB (1; 0.2%).

### 3.3. Prevalence of Integrase DRMs

Mutations associated with DR to INSTIs were evaluated in samples from treatment-naïve (ART-naïve) patients (*n* = 225) and patients with virological failure of therapy without INSTIs (INSTI-naïve) (*n* = 183).

Among ART-naïve patients, DRMs were detected in only 3 samples (1.3%). The major mutation, Q146P, was accompanied by the accessory mutation G163R in one patient and by itself in the second patient. The third patient had only the accessory mutation G163R. Thus, according to interpretation-predicted DR from the HIVdb Program, the first patient had high-level EVG and low-level RAL resistance, the second patient had high-level EVG resistance, and the third patient had low-level resistance to EVG and RAL. No patients in this group had mutations on the list of DRMs used for surveillance of transmitted HIV-1 DR (SDRM) [49].

DRMs were detected among 5 INSTI-naïve patients (2.7%). Two of the patients had major mutations, including R263K (1/183; 0.5%), which was associated with intermediate-level DTG, EVG resistance, and low-level RAL, BIC resistance, and unusual mutation in DR-position S147T, which does not reduce susceptibility to any INSTI. Additionally, 3 INSTI-naïve patients had accessory mutations: E157Q (2/183; 1.1%) and T97A (1/183; 0.5%), which alone have minimal impact on INSTI susceptibility or HIV-1 replication capacity. Only one mutation, R263K, was in the list of SDRMs.

### 3.4. Prevalence of Naturally Occurring Integrase Polymorphisms

**Figure 1:**
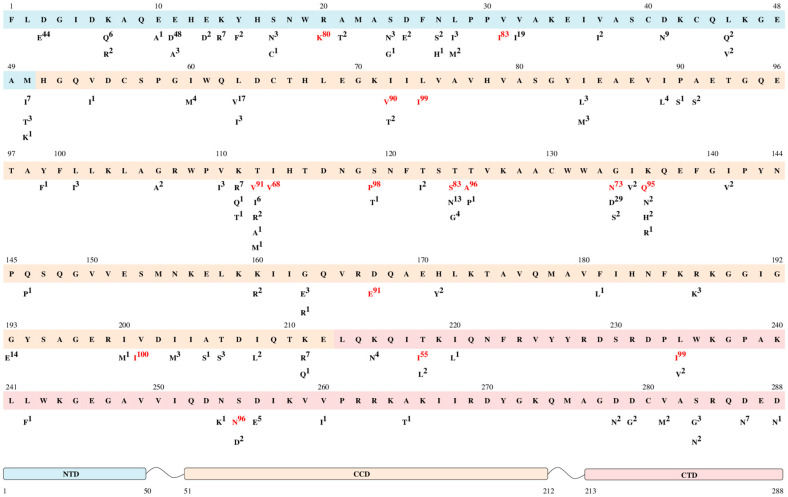
Frequency of naturally occurring polymorphisms of the A6 sub-subtype. The reference sequence (HXB2 HIV-1 clade B) is shown in the top line. Letters and numbers below each position are the polymorphisms and their frequency (%). Highly polymorphic mutations (more than 50% in A6-sequences) are indicated in red. The N-terminal domain (NTD) is indicated in blue at positions 1–50, the catalytic core domain (CCD) is indicated in orange at positions 51–212 and the C-terminal domain (CTD) is indicated in pink at positions 213–288.

Among the A6 clade (*n* = 193), for 75 of 288 (26.1%) at least one polymorphism was detected within integrase (Figure 1). A total of 108 mutations were determined in these polymorphic positions. The most polymorphic positions were identified in the N-terminal domain (20/50, 40%), none of which were in the conserved zinc finger motif. In the catalytic core domain, 39 polymorphic positions (39/162, 24.1%) were determined, all of which were out of the DDE catalytic triad. In the C-terminal domain, 16 polymorphic positions were identified (16/76, 21.1%). Additionally, the amino acids in integrase positions that were identified as critical for interaction with the essential HIV integration cofactor LEDGF/p75 linking integrase to chromatin (128, 130, 131, 161, 165, 166, 168, 170, 172, 173, 214, and 216) were determined to be conserved [9].

We determined 16 polymorphic mutations detected in more than 50% (highly polymorphic) of A6 sequences, which more often belong to the part of the catalytic core domain.

Five highly polymorphic mutations, R20K [50], I72V [9,33,51], L74I [7,23,24,25,26], S119P [51,52], and V201I [11,26,38,53], were frequently reported in regard to a small reduction in replication capacity relative to the wild type. It has been reported that in the absence of major mutations, all these polymorphic mutations had little, if any, effect on drug susceptibility in vitro, thus suggesting a secondary role for viral fitness rescue and increasing resistance.

The 9 amino acid substitutions (V31I [22,35,54], T112V [35,39,54], I113V [26], T125A [24,26,35,54], G134N [35,39,54], K136Q [39,54], D167E [22,35,54], T218I [22,54], and L234I [22,35,54]) were described as polymorphic mutations in non-B clades, none of which were ascribed to INSTI resistance.

Other mutations (T124S, S255N) have not been previously reported.

**Figure 2:**
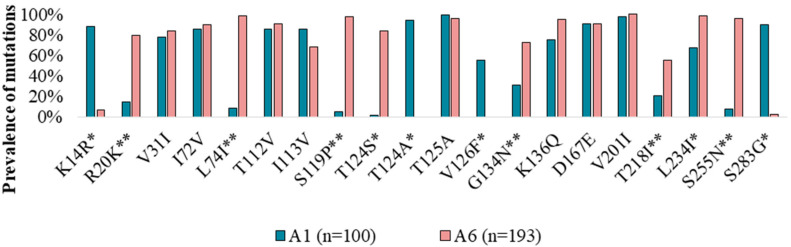
Prevalence of integrase highly polymorphic mutations in A1 and A6 viral clades from treatment-naïve patients. The statistically significant differences (*p* < 0.05) between A1 and A6 are indicated by *, and the unique A6 polymorphic mutations are indicated by **.

In addition, we analyzed highly polymorphic positions for the A1 sequences (*n* = 100) as the closest and most studied clade of subtype A. We compared positions that were highly polymorphic for at least one of the A6 or A1 clades and identified 19 amino acid positions and 20 substitutions in them (Figure 2, Table S2).

For the A6 and A1 clades, amino acids in 8 highly polymorphic positions (31, 72, 112, 113, 125, 136, 167, and 201) were similar between sub-subtypes and differed from the B consensus sequence.

In 11 positions (14, 20, 74, 119, 124, 126, 134, 218, 234, 255, and 283), A6 sequences were significantly different (*p* < 0.05) from the A1 clade, which can be used as a sub-subtype-specific marker to distinguish them.

In 6 positions (20, 74, 119, 134, 218, and 255), A6 had amino acids that differed from those of the A1 and B clades. Conversely, in 3 positions (14, 26, and 283), A1 sequences had highly polymorphic mutations, while A6 clades had no substitutions (like the B clade).

Interestingly, position 124 had different highly polymorphic mutations in the A1 (T124A) and A6 (T124S) sequences.

### 3.5. Genetic Barrier

Naturally occurring polymorphisms can affect the genetic barrier to the development of INSTI resistance, defined as the number of transitions or transversions required to overcome drug selection pressure [32,33]. Therefore, 32 integrase amino acid positions (50, 51, 54, 66, 68, 74, 92, 95, 97, 114, 118, 119, 121, 128, 138, 140, 142, 143, 145, 146, 147, 148, 149, 151, 153, 155, 157, 163, 193, 230, 232, and 263) related to 58 resistance mutations were analyzed for a genetic barrier (Table S3). In these positions, the frequency of occurrence of each codon was calculated. For codons occurring in these subtypes more often than 1%, the number of transversions (tv) and transitions (ts) necessary for a resistance substitution (resistant codon) was calculated.

A comparative analysis of the sub-subtypes A1 (*n* = 100), A6 (*n* = 193), and subtype B (*n* = 2577) sequences showed that the genetic barrier was similar at almost all positions.

For two positions (140 and 151), subtype A (A1 and A6) had a significantly different genetic barrier from subtype B (Table 1).

Position 140, in most sequences of A1 and A6, is encoded by GGG/GGA, and in subtype B, there is a GGC/GGT codon. These codons determine that, with respect to subtype B, the calculated barrier for the G140C mutation, which is a major mutation and associated with resistance to all INSTIs, was equal to 2.5, and a score of 5 was calculated for subtype A.

Position 151 in most sequences of subtype B was codon GTA, and codon GTG was present in sequences of subtype A. Therefore, for the V151I mutation, which could play a role in the resistance to INSTIs, for both sub-subtypes A1 and A6, the genetic barrier was also higher than that of subtype B (0.4 versus 0.2, respectively).

Additionally, the sequences of sub-subtype A6 were significantly different from those in A1 and B at position 74. At this position, for the vast majority of sequences of sub-subtype A6, the codon ATA was present, which determined the genetic barrier for L74M (score of 1) and L74I (score of 0). In most sequences of sub-subtype A1 and subtype B, this position was encoded by the CTG, which determined the genetic barrier to 74M and 74I with scores of 2.5 and 2.7, respectively. For position L74F, A6 sequences had a score of 5, while for sequences of A1 and B, the barrier had a score of 3.5.

### 3.6. Founder Effect of the L74I Mutation

The L74I mutation appears to be a key characteristic of sub-subtype A6. To investigate the origin of the mutation in this genetic variant, we performed phylogenetic analyses using A6 (*n* = 723), A (*n* = 56), A1 (*n* = 319), A2 (*n* = 7), A3 (*n* = 9), and A4 (*n* = 3) sequences from different geographic regions.

Comparative analysis of codon 74 among different genetic variants of subtype A showed that the frequency of substitutions was significantly different (*p* < 0.05) between the A6 clade and other sub-subtypes A. Sub-subtype A6 and other genetic variants of subtype A included L74L in 3.7% and 86.4% of cases, L74I in 94.9% and 9.6%, L74M in 1.2% and 3%, and L74V in 0.1% and 1%, respectively.

**Figure 3:**
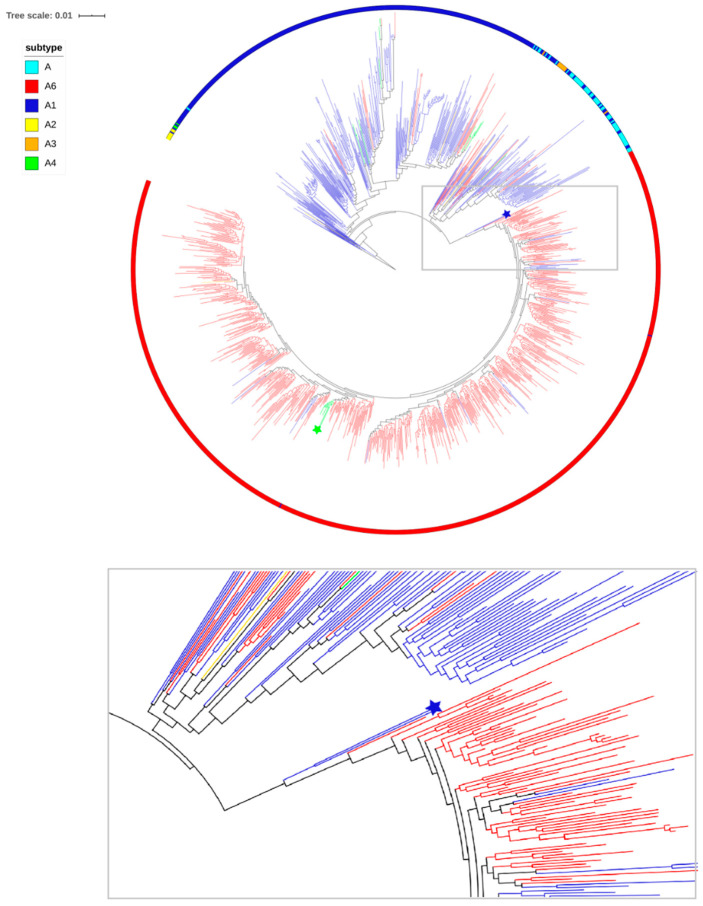
The maximum likelihood (ML) tree of the pol region sequences of subtype A included 723 tips of clade A6 and 394 tips of the other subtype A (A, A1, A2, A3, and A4). Subtypes are displayed on the outside of the tree (colored ring). Branches are colored according to the status of the 74 codon in the sequences: Blue, red, green, and yellow for sequences with L74L, L74I, L74M, and L74V, respectively. The blue star indicates A6.IT.2002.60000. EU861977 and A6.RU.2000.RU00051. EF545108 sequences. The green star indicates the cluster of viruses with the L74M mutation.

The phylogenetic tree is shown in Figure 3. Phylogenetic analysis suggests that A6 sequences formed a well-defined monophyletic subcluster among different sub-subtypes of subtype A. Within the A6 subcluster, there are a few branches with L74L, suggesting that in a few cases, L74I can revert to wild type. In addition to the majority of samples from Russia, clade A6 contains sequences from Uzbekistan, Tajikistan, Kazakhstan, Armenia, Ukraine, Georgia, and Belarus, 7 sequences from Cyprus, and single sequences from England and Italy.

Interestingly, there was a small separate cluster of viruses with L74M, including 7 samples from Uzbekistan, one sample from Russia (Tomsk), and one sample from Kuwait. However, analysis of the genetic distance between these samples showed that only 7 sequences from Uzbekistan differ by less than 1% (0.2–1.0%), which suggests an epidemiological link between the samples. 

Notably, two outlier sequences from Italy (A6.IT.2002.60000. EU861977) and Russia (A6.RU.2000.RU00051. EF545108) with L74L were placed close to the root of the A6 tree. These sequences can be considered as close links to the potential founder of the monophyletic clade of sub-subtype A6. The sequences with L74L belonged to heterosexuals, identified before the burst of the large epidemic among the injectors. Clearly, the viruses with the L74I mutation spread as a result of a founder effect among injectors across FSU countries.

Additionally, given that the majority of A1 sequences, which are the source of A6, do not have any substitutions in codon 74, the overall picture is that L74I was selected at the early stage of the epidemic among injectors in Russia and subsequently spread as a founder effect in Russia and other regions.

The phylodynamic analysis revealed that the time of the most recent common ancestor (tMRCA) for sub-subtype A6 in Russia was 1998 (95% HPD: 1989–2003). tMRCA is considered the approximate time of infection of the potential founder of the sub-subtype A6 epidemic, which was determined before its expansion among IDUs in Russia.

## 4. Discussion

The development and expanding use of INSTIs in ARV-naïve and ARV-experienced patients make it increasingly important to survey INSTI resistance. Moreover, this is particularly important for patients with non-B viruses because of the lack of data about features of genetic variants and virological outcomes of treatment in these patients.

In this study, we have described the genetic features of HIV-1 integrase sub-subtype A6, which dominated Russia and spread successfully across FSU countries and evolved into one of the fastest-growing epidemics in the world [39,40,41,42]. As expected, the most frequent viral genetic variant for our data samples was sub-subtype A6 (85.8%).

It should be noted that the dominant transmission routes for the studied patients were sexual contact (46.5%), and only 25% were in the traditional risk group (intravenous drug users), which is typical for the Russian epidemic since 2015.

Because the use of INSTIs was introduced recently in Russia [6], we found a low level of INSTI DR. Overall, the frequency of at least one INSTI resistance mutation was 1.3% and 2.7% in treatment-naïve and INSTI-naïve patients, respectively. SDRMs were detected only in 1 INSTI-naïve patient. These data suggest that the implementation of INSTI drugs for the treatment of HIV-infected ARV-naïve and ARV-experienced patients in Russia will be successful.

However, not only DRMs can affect sensitivity changes to drugs. Previous studies have shown that the virological outcomes of a therapy can be related to subtype-specific differences, pointing to the importance of analyzing the features of HIV subtypes for an improved understanding of viral subtype variability in the occurrence of DR [13,15,16,22,32,33,34,35,36,37,38].

Subtype-specific differences related to polymorphic mutations that can affect viral fitness and can influence INSTI efficacy, even in the absence of major DRMs, can be emerging threats to the success of cART.

Our data have added to the overall understanding of the prevalence of natural polymorphisms in A6 sub-subtype infections [18,19,20,21]. Overall, 26.1% of positions in integrase were defined as polymorphic. A total of 108 mutations were detected, including 16 highly polymorphic mutations. Five of them (R20K, I72V, L74I, S119P, and V201I) were described as INSTI resistant [7,9,11,23,24,25,26,33,36,49,50,51,52].

Remarkably, the prevalence of 12 polymorphic mutations (K14R, R20K, L74I, S119P, T124S, T124A, V126F, G134N, T218I, L234I, S255N, and S283G) was significantly different between sub-subtypes A6 and A1. This characteristic can be used as a marker of the A6 variant, allowing discrimination of this sub-subtype from A1 viruses. Taking into account that the A6 virus dominating Russia may be misclassified as A1, these gene features allow us to clarify the viral sub-subtype in samples and correct the data about the circulation of different A-subtype viruses.

Naturally occurring polymorphisms can also impact the genetic barrier to DR by influencing the selection of resistance mutations, enzymatic activity, and replicative capacity [12].

The genetic barrier, defined as the number of mutations required to overcome drug selective pressure, is one of the important factors in the development of HIV DR [32].

Analysis of genetic barriers among genetic variants A1, A6, and B displayed a similar genetic barrier for almost all positions, which is consistent with published data [32]. In a large number of sequences of subtype A, we also showed that subtype B differs from subtype A at positions 140 and 151, and subtype B had a lower genetic barrier to the occurrence of mutations G140C and V151I, which could play a role in INSTI resistance.

Additionally, for position 74, we showed a lower genetic barrier for L74M and L74I mutations in sub-subtype A6. This suggests that important differences at position 74 may lead to a lower genetic barrier for the drug resistance pathway in the A6 sub-subtype epidemics.

The L74I mutation appears to be a key characteristic of sub-subtype A6. Therefore, we estimated the temporal origin and phylogenetic characteristics of the sub-subtype A6 viruses with the L74I mutation and showed that viruses with L74I spread as a result of a founder effect among IDUs across FSU countries, validating our initial hypothesis.

The origin and dispersal of L74I are identical, and the tMRCA of L74I was estimated in 1998, which is in accordance with the previously estimated dates of the A6 epidemic among the injectors.

The findings of this article show the important role of features of viral genotypes in drug resistance and also indicate that there is still a need for a better understanding of resistance mechanisms to INSTIs. The potential contribution of naturally occurring polymorphisms, particularly the L74I mutation, in HIV-1 integrase to the evolution of resistance under the selective pressure of INSTIs may have clinical and virological implications. A lower genetic barrier for L74I mutations in sub-subtype A6 can lead to quicker development of drug resistance than subtype B, which is especially important in light of virological failures in patients with the L74I mutation from Russia in the ATLAS and FLAIR studies [15,16]. In vitro studies and studies in clinical practice are necessary to determine what facilitates INSTI resistance in non-B subtypes and how baseline polymorphic mutations impact clinical or virological outcomes.

## Figures and Tables

**Table 1:** The prevalence of codons in sequences A1, A6, and B and the number of mutations required to obtain resistant codons.

Position	Codon	A1 (*n* = 100)	A6 (*n* = 193)	B (*n* = 2577)	Resistant Codon	Mutation *	Resistant Codon	Mutation *	Resistant Codon	Mutation *
		Proportion			L74M		L74I		L74F	
74	CTG	**64%**	0%	**82%**	ATG	1 tv	ATA	1 tv, 1 ts (GA)	TTC/T	1 tv, 1 ts
	CTA	19%	<1%	4%	ATG	1 tv, 1 ts	ATA	1 tv	TTC/T	1 tv, 1 ts
	ATA	8%	**98%**	7%	ATG	1 ts	ATA	0	TTC/T	2 tv
	TTG	4%	0%	1%	ATG	1 tv	ATA	1 tv, 1 ts (GA)	TTC/T	1 tv
	ATG	2%	0%	<1%	ATG	0	ATA	1 ts (GA)	TTC/T	2 tv
	GTG	0%	0%	3%	ATG	1 ts	ATA	2 ts (GA)	TTC/T	2 tv
140	codon	Proportion			G140A		G140C		G140S	
	GGC	0%	0%	**81%**	GCC	1 tv	TGC	1 tv	TCC	2 tv
	GGT	0%	0%	13%	GCT	1 tv	TGT	1 tv	TCT	2 tv
	GGA	47%	11%	3%	GCA	1 tv	TGC/T	2 tv	TCA	2 tv
	GGG	**52%**	**85%**	2%	GCG	1 tv	TGC/T	2 tv	TCG	2 tv
	GGR	1%	4%	<1%	GCA/G	1 tv	TGC/T	2 tv	TCA/G	2 tv
151	codon	Proportion			V151A		V151I		V151L	
	GTA	14%	6%	**94%**	GCA	1 ts	ATA	1 ts (GA)	CTA	1 tv
	GTG	**86%**	**89%**	4%	GCG	1 ts	ATA	2 ts (GA)	CTG	1 tv
	ATA	0%	0%	1%	GCA	2 ts	ATA	0	CTA	1 tv
	GTR	0%	4%	<1%	GCA/G	1 ts	ATA	1/2 ts (GA)	CTA/G	1 tv

* tv indicates transversion, ts indicates transition. The numbers in boldface are value is more than 50%.

## Supplementary Materials

The following are available online at https://www.mdpi.com/1999-4915/12/8/838/s1, Table S1: Characteristics of the patients, Table S2: The prevalence of integrase highly polymorphic mutations in A1 and A6 viral clades in treatment-naïve patients, Table S3: The prevalence of codons in sequences A1, A6, and B and the number of mutations required to obtain resistant codons.
