# Supplementary material for: Genetic Features of HIV-1 Integrase Sub-Subtype A6 Predominant in Russia and Predicted Susceptibility to INSTIs
Source: Viruses. 2020 Jul 31;12(8):838. doi: 10.3390/v12080838 (PMC7472261; doi:10.3390/v12080838)
Supplement: Supplementary file 1 [file viruses-12-00838-s001.zip › viruses-876257-supplementary/Table 1 of Supplementary Materials.docx]

**Table S1. Characteristics of the patients**

| Characteristic |  | **Overall (N=408)** | **Treatment-naïve patients (N=225)** | **INSTI-naïve patients (N=183)** |
| --- | --- | --- | --- | --- |
| Age (years), median (IQR) | | 32 (26-40) | 30 (25-38) | 36 (30-41) |
| Sex, n (%) |  |  |  |  |
|  | Male | 235 (57.6) | 131 (58.2) | 104 (56.8) |
|  | Female | 173 (42.4) | 94 (41.8) | 79 (43.2) |
| Route of HIV transmission, n (%) | |  |  |  |
|  | Sexual | 212 (52.0) | 131 (58.2) | 81 (44.3) |
|  | Heterosexual | 167 (40.9) | 108 (48.0) | 59 (32.2) |
|  | MSM | 23 (5.6) | 21 (9.3) | 2 (1.1) |
|  | IDU | 102 (25.0) | 42 (18.7) | 60 (32.8) |
|  | MTCT | 20 (4.9) | 4 (1.8) | 16 (8.7) |
|  | Outbreak | 4 (1.0) | 1 (0.4) | 3 (1.6) |
|  | Unknown | 70 (17.2) | 47 (20.9) | 23 (12.6) |
| Region, n (%) | |  |  |  |
|  | Bryansk | 12 (2.9) | 11 (4.9) | 1 (0.5) |
|  | Irkutsk | 1 (0.2) | 0 | 1 (0.5) |
|  | Ivanovo | 4 (1.0) | 2 (0.9) | 2 (1.1) |
|  | Kaluga | 1 (0.2) | 0 | 1 (0.5) |
|  | Khanty-Mansiysk | 11 (2.7) | 11 (4.9) | 0 |
|  | Kostroma | 6 (1.5) | 0 | 6 (3.3) |
|  | Krasnodar | 89 (21.8) | 71 (31.6) | 18 (9.8) |
|  | Krasnoyarsk | 12 (2.9) | 11 (4.9) | 1 (0.5) |
|  | Lipetsk | 16 (3.9) | 16 (7.1) | 0 |
|  | Moscow | 22 (5.4) | 19 (8.4) | 3 (1.6) |
|  | Orenburg | 1 (0.2) | 0 | 1 (0.5) |
|  | Oryol | 58 (14.2) | 4 (1.8) | 54 (29.5) |
|  | Perm | 2 (0.5) | 0 | 2 (1.1) |
|  | Republic of Buryatia | 21 (5.1) | 16 (7.1) | 5 (2.7) |
|  | Republic of Khakassia | 3 (0.7) | 0 | 3 (1.6) |
|  | Ryazan | 2 (0.5) | 0 | 2 (1.1) |
|  | Samara | 57 (14.0) | 28 (12.4) | 29 (15.8) |
|  | Smolensk | 9 (2.2) | 9 (4.0) | 0 |
|  | Stavropol | 1 (0.2) | 1 (0.4) | 0 |
|  | Tambov | 4 (1.0) | 0 | 4 (2.2) |
|  | Tula | 30 (7.4) | 0 | 30 (16.4) |
|  | Tver | 38 (9.3) | 26 (11.6) | 12 (6.6) |
|  | Vladimir | 1 (0.2) | 0 | 1 (0.5) |
|  | Yaroslavl | 7 (1.7) | 0 | 7 (3.8) |
| Federal district, n (%) | |  |  |  |
|  | Central | 209 (51.2) | 87 (38.7) | 122 (66.7) |
|  | Far Eastern | 18 (4.4) | 15 (6.7) | 3 (1.6) |
|  | North Caucasian | 1 (0.2) | 1 (0.4) | 0 |
|  | Siberian | 20 (4.9) | 12 (5.3) | 8 (4.4) |
|  | Southern | 89 (21.8) | 71 (31.6) | 18 (9.8) |
|  | Ural | 11 (2.7) | 11 (4.9) | 0 |
|  | Volga | 60 (14.7) | 28 (12.4) | 32 (17.5) |
| Date of first positive immunoblot, n (%) | |  |  |  |
|  | 1999 | 3 (0.7) | 1 (0.4) | 2 (1.1) |
|  | 2000 | 11 (2.7) | 2 (0.9) | 9 (4.9) |
|  | 2001 | 16 (3.9) | 3 (1.3) | 13 (7.1) |
|  | 2002 | 6 (1.5) | 1 (0.4) | 5 (2.7) |
|  | 2003 | 8 (2.0) | 1 (0.4) | 7 (3.8) |
|  | 2004 | 8 (1.5) | 2 (0.9) | 4 (2.2) |
|  | 2005 | 4 (1.0) | 1 (0.4) | 3 (1.6) |
|  | 2006 | 9 (2.2) | 0 | 9 (4.9) |
|  | 2007 | 17 (4.2) | 15 (6.7) | 2 (1.1) |
|  | 2008 | 17 (4.2) | 3 (1.3) | 14 (7.7) |
|  | 2009 | 13 (3.2) | 5 (2.2) | 8 (4.4) |
|  | 2010 | 14 (3.4) | 3 (1.3) | 11 (6.0) |
|  | 2011 | 21 (5.1) | 6 (2.7) | 15 (8.2) |
|  | 2012 | 13 (3.2) | 4 (1.8) | 9 (4.9) |
|  | 2013 | 22 (5.4) | 11 (4.9) | 11 (6.0) |
|  | 2014 | 48 (11.8) | 37 (16.4) | 11 (6.0) |
|  | 2015 | 57 (14.0) | 46 (20.4) | 11 (6.0) |
|  | 2016 | 44 (10.8) | 33 (14.7) | 11 (6.0) |
|  | 2017 | 53 (13.0) | 44 (19.6) | 9 (4.9) |
|  | 2018 | 5 (1.2) | 5 (2.2) | 0 |
| Viral load category (log_10_ copies/mL)*, n (%) | |  |  |  |
|  | <2.7 | 15 (3.7) | 4 (1.8) | 11 (6.0) |
|  | 2.7-3 | 18 (4.4) | 5 (2.2) | 13 (7.1) |
|  | 3-4 | 78 (19.1) | 37 (16.4) | 41 (22.4) |
|  | 4-5 | 131 (32.1) | 85 (37.8) | 46 (25.1) |
|  | >5 | 87 (21.3) | 49 (21.8) | 38 (20.8) |
| Viral load (log_10_ copies/mL)*, median (IQR) | | 4.4 (4.0-5.0) | 4.1 (3.3-5.0) | 4.4 (3.7-5.0) |
| CD4+ T-cell count (cells/mm3)*, median (IQR) | | 314 (158-553) | 445,5 (291-646) | 225 (97-410) |
| Baseline regimen, n (%) | |  |  |  |
|  | NRTI | 180 (44.1) | 0 | 180 (98.4) |
|  | NNRTI | 116 (28.4) | 0 | 116 (63.4) |
|  | PI | 103 (25.2) | 0 | 103 (56.3) |
|  | INSTI | 0 | 0 | 0 |
| Subtype, n (%) |  |  |  |  |
|  | A1 | 1 (0.2) | 0 | 1 (0.5) |
|  | A6 | 350 (85.8) | 193 (85.8) | 157(85.8) |
|  | B | 29 (7.1) | 16 (7.1) | 13 (7.1) |
|  | G | 3 (0.7) | 3 (1.3) | 0 |
|  | CRF02_AG | 10 (2.5) | 4 (1.8) | 6 (3.3) |
|  | CRF03_AB | 1 (0.2) | 0 | 1 (0.5) |
|  | CRF63_02A1 | 14 (3.4) | 9 (4.0) | 5 (2.7) |

* for patients with test results obtained no more than 3 months prior to the genotyping test
