# Supplementary material for: Genetic Features of HIV-1 Integrase Sub-Subtype A6 Predominant in Russia and Predicted Susceptibility to INSTIs
Source: Viruses. 2020 Jul 31;12(8):838. doi: 10.3390/v12080838 (PMC7472261; doi:10.3390/v12080838)
Supplement: Supplementary file 1 [file viruses-12-00838-s001.zip › viruses-876257-supplementary/Table 2 of Supplementary Materials.docx]

| Polymorphic mutations | A1 (n=100) | A6 (n=193) | P value |
| --- | --- | --- | --- |
| K14R | 88.0% | 6.8% | <0.001 |
| R20K | 15.0% | 79.8% | <0.001 |
| V31I | 78.0% | 83.4% | 0.717 |
| I72V | 86.0% | 89.6% | 0.819 |
| L74I | 9.0% | 99.0% | <0.001 |
| T112V | 86.0% | 90.7% | 0.770 |
| I113V | 86.0% | 68.4% | 0.217 |
| S119P | 5.0% | 97.9% | <0.001 |
| T124S | 2.0% | 83.4% | <0.001 |
| T124A | 94.0% | 0.0% | <0.001 |
| T125A | 99.0% | 96.4% | 0.878 |
| V126F | 55.0% | 0.0% | <0.001 |
| G134N | 31.0% | 72.5% | <0.001 |
| K136Q | 75.0% | 94.8% | 0.203 |
| D167E | 91.0% | 91.2% | 0.991 |
| V201I | 98.0% | 100.0% | 0.909 |
| T218I | 21.0% | 55.4% | <0.001 |
| L234I | 67.0% | 99.0% | 0.038 |
| S255N | 8.0% | 96.4% | <0.001 |
| S283G | 90.0% | 2.6% | <0.001 |

**Table S2. The prevalence of integrase highly polymorphic mutations in A1 and A6 viral clades in treatment-naïve patients**
