# Supplementary material for: Genetic Features of HIV-1 Integrase Sub-Subtype A6 Predominant in Russia and Predicted Susceptibility to INSTIs
Source: Viruses. 2020 Jul 31;12(8):838. doi: 10.3390/v12080838 (PMC7472261; doi:10.3390/v12080838)
Supplement: Supplementary file 1 [file viruses-12-00838-s001.zip › viruses-876257-supplementary/Table 3 of Supplementary Materials.docx]

**Table S3. The prevalence of codons in sequences A1, A6 and B and the number of mutations required to obtain resistant codons.**

| Substitution | Genetic barrier score | Number of sequences | | | | Proportion, % | | |
| --- | --- | --- | --- | --- | --- | --- | --- | --- |
|  |  | A1 | A6 | B | A1 | | A6 | B |
| M50I | -2331 | 0 | 0 | 1 | 0.00 | | 0.00 | 0.04 |
|  | 0 | 10 | 13 | 378 | 10.00 | | 6.74 | 14.67 |
|  | 0.2 | 85 | 174 | 2153 | **85.00** | | **90.16** | **83.55** |
|  | 0.4 | 1 | 0 | 3 | 1.00 | | 0.00 | 0.12 |
|  | 1 | 1 | 0 | 17 | 1.00 | | 0.00 | 0.66 |
|  | 1.2 | 2 | 4 | 14 | 2.00 | | 2.07 | 0.54 |
|  | 2.5 | 0 | 0 | 3 | 0.00 | | 0.00 | 0.12 |
|  | 2.7 | 1 | 2 | 8 | 1.00 | | 1.04 | 0.31 |
| H51Y | 1 | 100 | 193 | 2569 | **100.00** | | **100.00** | **99.69** |
|  | 1.2 | 0 | 0 | 4 | 0.00 | | 0.00 | 0.16 |
|  | 2.5 | 0 | 0 | 1 | 0.00 | | 0.00 | 0.04 |
|  | 3.5 | 0 | 0 | 3 | 0.00 | | 0.00 | 0.12 |
| V54I | 0 | 0 | 2 | 8 | 0.00 | | 1.04 | 0.31 |
|  | 0.2 | 98 | 187 | 2530 | **98.00** | | **96.89** | **98.18** |
|  | 0.4 | 2 | 4 | 37 | 2.00 | | 2.07 | 1.44 |
|  | 1.2 | 0 | 0 | 2 | 0.00 | | 0.00 | 0.08 |
| T66A | 0 | 0 | 0 | 1 | 0.00 | | 0.00 | 0.04 |
|  | 1 | 100 | 193 | 2571 | **100.00** | | **100.00** | **99.77** |
|  | 2 | 0 | 0 | 3 | 0.00 | | 0.00 | 0.12 |
|  | 2.5 | 0 | 0 | 2 | 0.00 | | 0.00 | 0.08 |
| T66I | 0 | 0 | 0 | 4 | 0.00 | | 0.00 | 0.16 |
|  | 1 | 99 | 192 | 2556 | **99.00** | | **99.48** | **99.19** |
|  | 1.2 | 1 | 1 | 15 | 1.00 | | 0.52 | 0.58 |
|  | 3.5 | 0 | 0 | 2 | 0.00 | | 0.00 | 0.08 |
| T66K | 2.5 | 99 | 190 | 2483 | **99.00** | | **98.45** | **96.35** |
|  | 2.7 | 0 | 0 | 1 | 0.00 | | 0.00 | 0.04 |
|  | 5 | 1 | 3 | 93 | 1.00 | | 1.55 | 3.61 |
| L68V | 0 | 0 | 0 | 66 | 0.00 | | 0.00 | 2.56 |
|  | 1 | 1 | 1 | 19 | 1.00 | | 0.52 | 0.74 |
|  | 2.5 | 99 | 192 | 2487 | **99.00** | | **99.48** | **96.51** |
|  | 3.5 | 0 | 0 | 4 | 0.00 | | 0.00 | 0.16 |
|  | 5 | 0 | 0 | 1 | 0.00 | | 0.00 | 0.04 |
| L74M | -2331 | 0 | 0 | 1 | 0.00 | | 0.00 | 0.04 |
|  | 0 | 2 | 0 | 23 | 2.00 | | 0.00 | 0.89 |
|  | 0.2 | 1 | 0 | 70 | 1.00 | | 0.00 | 2.72 |
|  | 1 | 8 | 190 | 186 | 8.00 | | **98.45** | 7.22 |
|  | 2.5 | 70 | 1 | 2171 | **70.00** | | 0.52 | **84.25** |
|  | 3.5 | 19 | 2 | 123 | 19.00 | | 1.04 | 4.77 |
|  | 5 | 0 | 0 | 3 | 0.00 | | 0.00 | 0.12 |
| L74I | -2331 | 0 | 0 | 1 | 0.00 | | 0.00 | 0.04 |
|  | 0 | 8 | 191 | 187 | 8.00 | | **98.96** | 7.26 |
|  | 0.2 | 2 | 0 | 24 | 2.00 | | 0.00 | 0.93 |
|  | 0.4 | 1 | 0 | 68 | 1.00 | | 0.00 | 2.64 |
|  | 2.5 | 20 | 2 | 133 | 20.00 | | 1.04 | 5.16 |
|  | 2.7 | 69 | 0 | 2162 | **69.00** | | 0.00 | **83.90** |
|  | 5 | 0 | 0 | 2 | 0.00 | | 0.00 | 0.08 |
| L74F | -2331 | 0 | 0 | 1 | 0.00 | | 0.00 | 0.04 |
|  | 1 | 0 | 0 | 2 | 0.00 | | 0.00 | 0.08 |
|  | 2.5 | 6 | 2 | 56 | 6.00 | | 1.04 | 2.17 |
|  | 3.5 | 83 | 1 | 2241 | **83.00** | | 0.52 | **86.96** |
|  | 5 | 11 | 190 | 277 | 11.00 | | **98.45** | 10.75 |
| E92Q | 0 | 0 | 0 | 1 | 0.00 | | 0.00 | 0.04 |
|  | 2.5 | 100 | 193 | 2574 | **100.00** | | **100.00** | **99.88** |
|  | 2.7 | 0 | 0 | 2 | 0.00 | | 0.00 | 0.08 |
| E92G | 0 | 0 | 0 | 4 | 0.00 | | 0.00 | 0.16 |
|  | 1 | 100 | 193 | 2573 | **100.00** | | **100.00** | **99.84** |
| E92V | 2.5 | 100 | 193 | 2577 | **100.00** | | **100.00** | **100.00** |
| Q95K | -2331 | 0 | 0 | 1 | 0.00 | | 0.00 | 0.04 |
|  | 2.5 | 99 | 192 | 2562 | **99.00** | | **99.48** | **99.42** |
|  | 2.7 | 0 | 0 | 4 | 0.00 | | 0.00 | 0.16 |
|  | 5 | 1 | 1 | 10 | 1.00 | | 0.52 | 0.39 |
| T97A | 0 | 7 | 0 | 19 | 7.00 | | 0.00 | 0.74 |
|  | 1 | 92 | 193 | 2556 | **92.00** | | **100.00** | **99.19** |
|  | 2.5 | 1 | 0 | 2 | 1.00 | | 0.00 | 0.08 |
| H114Y | 1 | 100 | 193 | 2576 | **100.00** | | **100.00** | **99.96** |
|  | 3.5 | 0 | 0 | 1 | 0.00 | | 0.00 | 0.04 |
| G118R | 0.2 | 3 | 3 | 45 | 3.00 | | 1.55 | 1.75 |
|  | 2.5 | 97 | 190 | 2532 | **97.00** | | **98.45** | **98.25** |
| S119R | -2331 | 0 | 0 | 3 | 0.00 | | 0.00 | 0.12 |
|  | 0 | 0 | 0 | 268 | 0.00 | | 0.00 | 10.40 |
|  | 0.2 | 0 | 0 | 1 | 0.00 | | 0.00 | 0.04 |
|  | 1 | 0 | 0 | 2 | 0.00 | | 0.00 | 0.08 |
|  | 2.5 | 94 | 189 | 2140 | **94.00** | | **97.93** | **83.04** |
|  | 3.5 | 0 | 1 | 44 | 0.00 | | 0.52 | 1.71 |
|  | 5 | 6 | 3 | 119 | 6.00 | | 1.55 | 4.62 |
| F121Y | 0.2 | 1 | 0 | 0 | 1.00 | | 0.00 | 0.00 |
|  | 2.5 | 99 | 192 | 2577 | **99.00** | | **99.48** | **100.00** |
|  | 3.5 | 0 | 1 | 0 | 0.00 | | 0.52 | 0.00 |
| A128T | 0 | 0 | 0 | 23 | 0.00 | | 0.00 | 0.89 |
|  | 0.2 | 100 | 193 | 2551 | **100.00** | | **100.00** | **98.99** |
|  | 1.2 | 0 | 0 | 2 | 0.00 | | 0.00 | 0.08 |
|  | 2.5 | 0 | 0 | 1 | 0.00 | | 0.00 | 0.04 |
| E138A | 2.5 | 100 | 193 | 2563 | **100.00** | | **100.00** | **99.46** |
|  | 3.5 | 0 | 0 | 14 | 0.00 | | 0.00 | 0.54 |
| E138K | 0 | 0 | 0 | 15 | 0.00 | | 0.00 | 0.58 |
|  | 0.2 | 100 | 193 | 2534 | **100.00** | | **100.00** | **98.33** |
|  | 2.7 | 0 | 0 | 28 | 0.00 | | 0.00 | 1.09 |
| E138T | 2.5 | 0 | 0 | 15 | 0.00 | | 0.00 | 0.58 |
|  | 2.7 | 100 | 193 | 2562 | **100.00** | | **100.00** | **99.42** |
| G140A | 0 | 0 | 0 | 1 | 0.00 | | 0.00 | 0.04 |
|  | 2.5 | 100 | 193 | 2574 | **100.00** | | **100.00** | **99.88** |
|  | 3.5 | 0 | 0 | 2 | 0.00 | | 0.00 | 0.08 |
| G140C | 2.5 | 0 | 1 | 2452 | 0.00 | | 0.52 | **95.15** |
|  | 5 | 100 | 192 | 125 | **100.00** | | **99.48** | 4.85 |
| G140S | 0 | 0 | 0 | 2 | 0.00 | | 0.00 | 0.08 |
|  | 0.2 | 0 | 1 | 2450 | 0.00 | | 0.52 | **95.07** |
|  | 2.5 | 0 | 0 | 1 | 0.00 | | 0.00 | 0.04 |
|  | 2.7 | 100 | 192 | 124 | **100.00** | | **99.48** | 4.81 |
| P142T | -2331 | 0 | 0 | 1 | 0.00 | | 0.00 | 0.04 |
|  | 2.5 | 100 | 193 | 2576 | **100.00** | | **100.00** | **99.96** |
| Y143C | 0 | 0 | 0 | 1 | 0.00 | | 0.00 | 0.04 |
|  | 1 | 100 | 193 | 2574 | **100.00** | | **100.00** | **99.88** |
|  | 2 | 0 | 0 | 2 | 0.00 | | 0.00 | 0.08 |
| Y143R | 1 | 0 | 0 | 3 | 0.00 | | 0.00 | 0.12 |
|  | 2 | 100 | 193 | 2574 | **100.00** | | **100.00** | **99.88** |
| Y143H | 0 | 0 | 0 | 2 | 0.00 | | 0.00 | 0.08 |
|  | 1 | 100 | 193 | 2575 | **100.00** | | **100.00** | **99.92** |
| Y143K | 2.5 | 0 | 0 | 1 | 0.00 | | 0.00 | 0.04 |
|  | 5 | 100 | 193 | 2576 | **100.00** | | **100.00** | **99.96** |
| Y143S | 1 | 0 | 0 | 1 | 0.00 | | 0.00 | 0.04 |
|  | 2.5 | 100 | 193 | 2574 | **100.00** | | **100.00** | **99.88** |
|  | 3.5 | 0 | 0 | 2 | 0.00 | | 0.00 | 0.08 |
| Y143G | 2 | 0 | 0 | 1 | 0.00 | | 0.00 | 0.04 |
|  | 2.5 | 0 | 0 | 1 | 0.00 | | 0.00 | 0.04 |
|  | 3.5 | 100 | 193 | 2575 | **100.00** | | **100.00** | **99.92** |
| Y143A | 3.5 | 0 | 0 | 1 | 0.00 | | 0.00 | 0.04 |
|  | 5 | 100 | 193 | 2576 | **100.00** | | **100.00** | **99.96** |
| P145S | 0 | 0 | 0 | 1 | 0.00 | | 0.00 | 0.04 |
|  | 1 | 100 | 193 | 2575 | **100.00** | | **100.00** | **99.92** |
|  | 2.5 | 0 | 0 | 1 | 0.00 | | 0.00 | 0.04 |
| Q146P | 0 | 0 | 2 | 0 | 0.00 | | 1.04 | 0.00 |
|  | 1 | 0 | 0 | 1 | 0.00 | | 0.00 | 0.04 |
|  | 2.5 | 100 | 191 | 2576 | **100.00** | | **98.96** | **99.96** |
| S147G | 0 | 0 | 0 | 1 | 0.00 | | 0.00 | 0.04 |
|  | 1 | 100 | 193 | 2576 | **100.00** | | **100.00** | **99.96** |
| Q148H | 0 | 0 | 0 | 1 | 0.00 | | 0.00 | 0.04 |
|  | 2.5 | 100 | 193 | 2575 | **100.00** | | **100.00** | **99.92** |
|  | 5 | 0 | 0 | 1 | 0.00 | | 0.00 | 0.04 |
| Q148K | 2.5 | 100 | 193 | 2576 | **100.00** | | **100.00** | **99.96** |
|  | 5 | 0 | 0 | 1 | 0.00 | | 0.00 | 0.04 |
| Q148R | 0 | 0 | 0 | 2 | 0.00 | | 0.00 | 0.08 |
|  | 1 | 100 | 193 | 2574 | **100.00** | | **100.00** | **99.88** |
|  | 2.5 | 0 | 0 | 1 | 0.00 | | 0.00 | 0.04 |
| Q148N | 2.5 | 0 | 0 | 1 | 0.00 | | 0.00 | 0.04 |
|  | 5 | 100 | 193 | 2575 | **100.00** | | **100.00** | **99.92** |
|  | 7.5 | 0 | 0 | 1 | 0.00 | | 0.00 | 0.04 |
| G149A | 0 | 0 | 0 | 3 | 0.00 | | 0.00 | 0.12 |
|  | 2.5 | 100 | 193 | 2573 | **100.00** | | **100.00** | **99.84** |
|  | 3.5 | 0 | 0 | 1 | 0.00 | | 0.00 | 0.04 |
| V151I | -777 | 0 | 0 | 1 | 0.00 | | 0.00 | 0.04 |
|  | 0 | 0 | 1 | 43 | 0.00 | | 0.52 | 1.67 |
|  | 0.2 | 14 | 20 | 2438 | 14.00 | | 10.36 | **94.61** |
|  | 0.4 | 86 | 172 | 94 | **86.00** | | **89.12** | 3.65 |
|  | 2.5 | 0 | 0 | 1 | 0.00 | | 0.00 | 0.04 |
| V151 L | -777 | 0 | 0 | 1 | 0.00 | | 0.00 | 0.04 |
|  | 0 | 0 | 0 | 2 | 0.00 | | 0.00 | 0.08 |
|  | 2.5 | 100 | 193 | 2573 | **100.00** | | **100.00** | **99.84** |
|  | 5 | 0 | 0 | 1 | 0.00 | | 0.00 | 0.04 |
| V151A | -777 | 0 | 0 | 1 | 0.00 | | 0.00 | 0.04 |
|  | 1 | 100 | 193 | 2541 | **100.00** | | **100.00** | **98.60** |
|  | 2 | 0 | 0 | 34 | 0.00 | | 0.00 | 1.32 |
|  | 3.5 | 0 | 0 | 1 | 0.00 | | 0.00 | 0.04 |
| S153Y | 2.5 | 96 | 191 | 2478 | **96.00** | | **98.96** | **96.16** |
|  | 3.5 | 0 | 0 | 1 | 0.00 | | 0.00 | 0.04 |
|  | 5 | 4 | 2 | 98 | 4.00 | | 1.04 | 3.80 |
| S153F | 0 | 0 | 0 | 1 | 0.00 | | 0.00 | 0.04 |
|  | 1 | 96 | 191 | 2478 | **96.00** | | **98.96** | **96.16** |
|  | 3.5 | 4 | 2 | 98 | 4.00 | | 1.04 | 3.80 |
| N155H | 2.5 | 100 | 193 | 2577 | **100.00** | | **100.00** | **100.00** |
| N155S | 0 | 0 | 0 | 1 | 0.00 | | 0.00 | 0.04 |
|  | 1 | 100 | 193 | 2576 | **100.00** | | **100.00** | **99.96** |
| N155T | 2.5 | 100 | 193 | 2577 | **100.00** | | **100.00** | **100.00** |
| N155D | 1 | 100 | 193 | 2577 | **100.00** | | **100.00** | **100.00** |
| E157Q | 0 | 3 | 0 | 164 | 3.00 | | 0.00 | 6.36 |
|  | 2.5 | 97 | 193 | 2406 | **97.00** | | **100.00** | **93.36** |
|  | 2.7 | 0 | 0 | 1 | 0.00 | | 0.00 | 0.04 |
|  | 5 | 0 | 0 | 6 | 0.00 | | 0.00 | 0.23 |
| G163R | -2331 | 0 | 0 | 1 | 0.00 | | 0.00 | 0.04 |
|  | 0 | 0 | 2 | 8 | 0.00 | | 1.04 | 0.31 |
|  | 0.2 | 95 | 187 | 2346 | **95.00** | | **96.89** | **91.04** |
|  | 1 | 0 | 0 | 20 | 0.00 | | 0.00 | 0.78 |
|  | 1.2 | 0 | 4 | 141 | 0.00 | | 2.07 | 5.47 |
|  | 2.5 | 5 | 0 | 40 | 5.00 | | 0.00 | 1.55 |
|  | 2.7 | 0 | 0 | 16 | 0.00 | | 0.00 | 0.62 |
|  | 3.5 | 0 | 0 | 5 | 0.00 | | 0.00 | 0.19 |
| G163K | -2331 | 0 | 0 | 1 | 0.00 | | 0.00 | 0.04 |
|  | 0 | 0 | 0 | 12 | 0.00 | | 0.00 | 0.47 |
|  | 0.2 | 0 | 7 | 158 | 0.00 | | 3.63 | 6.13 |
|  | 0.4 | 95 | 186 | 2336 | **95.00** | | **96.37** | **90.65** |
|  | 2.5 | 1 | 0 | 19 | 1.00 | | 0.00 | 0.74 |
|  | 2.7 | 0 | 0 | 22 | 0.00 | | 0.00 | 0.85 |
|  | 2.9 | 4 | 0 | 29 | 4.00 | | 0.00 | 1.13 |
| G193E | 0 | 2 | 26 | 201 | 2.00 | | 13.47 | 7.80 |
|  | 0.2 | 96 | 167 | 2324 | **96.00** | | **86.53** | **90.18** |
|  | 1 | 0 | 0 | 1 | 0.00 | | 0.00 | 0.04 |
|  | 1.2 | 2 | 0 | 17 | 2.00 | | 0.00 | 0.66 |
|  | 2.5 | 0 | 0 | 12 | 0.00 | | 0.00 | 0.47 |
|  | 2.7 | 0 | 0 | 22 | 0.00 | | 0.00 | 0.85 |
| S230R | 1 | 0 | 0 | 1 | 0.00 | | 0.00 | 0.04 |
|  | 2.5 | 100 | 193 | 2287 | **100.00** | | **100.00** | **88.75** |
|  | 3.5 | 0 | 0 | 289 | 0.00 | | 0.00 | 11.21 |
| D232N | 0 | 0 | 0 | 5 | 0.00 | | 0.00 | 0.19 |
|  | 0.2 | 100 | 193 | 2484 | **100.00** | | **100.00** | **96.39** |
|  | 0.4 | 0 | 0 | 3 | 0.00 | | 0.00 | 0.12 |
|  | 2.7 | 0 | 0 | 85 | 0.00 | | 0.00 | 3.30 |
| R263K | 0.2 | 100 | 193 | 2574 | **100.00** | | **100.00** | **99.88** |
|  | 0.4 | 0 | 0 | 1 | 0.00 | | 0.00 | 0.04 |
|  | 2.7 | 0 | 0 | 2 | 0.00 | | 0.00 | 0.08 |
